# Effect of DNA Glycosylases OGG1 and Neil1 on Oxidized G-Rich Motif in the *KRAS* Promoter

**DOI:** 10.3390/ijms22031137

**Published:** 2021-01-24

**Authors:** Annalisa Ferino, Luigi E. Xodo

**Affiliations:** Laboratory of Biochemistry, Department of Medicine, P.le Kolbe 4, 33100 Udine, Italy; annalisa.ferino@uniud.it

**Keywords:** G4 DNA, OGG1, Neil1, 8-oxoguanine, *KRAS*

## Abstract

The promoter of the *Kirsten ras* (*KRAS*) proto-oncogene contains, upstream of the transcription start site, a quadruplex-forming motif called 32R with regulatory functions. As guanine under oxidative stress can be oxidized to 8-oxoguanine (8OG), we investigated the capacity of glycosylases 8-oxoguanine glycosylase (OGG1) and endonuclease VIII-like 1 (Neil1) to excise 8OG from 32R, either in duplex or G-quadruplex (G4) conformation. We found that OGG1 efficiently excised 8OG from oxidized 32R in duplex but not in G4 conformation. By contrast, glycosylase Neil1 showed more activity on the G4 than the duplex conformation. We also found that the excising activity of Neil1 on folded 32R depended on G4 topology. Our data suggest that Neil1, besides being involved in base excision repair pathway (BER), could play a role on *KRAS* transcription.

## 1. Introduction

Reactive oxygen species (ROS) are byproducts of cellular metabolism and have a complex behavior in cancer cells [1]. At low levels, ROS act as signaling molecules promoting various pathways including proliferation, survival, angiogenesis, and metastasis [2,3,4]. At high levels, ROS mediate a cellular response leading to apoptosis and/or necrosis [5]. As cancer cells produce higher amounts of ROS than normal cells [6,7,8], they have developed an efficient mechanism for controlling the redox homeostasis. It involves Nrf2, a redox sensor protein that responds to oxidative stress and the thiol status of the cells [9]. When ROS increase, Nrf2 migrates into the nucleus, binds to the antioxidant response element (ARE), and activates a detoxification program that reduces the ROS level, thus favoring proliferation and survival [10,11]. The most common ROS sources are the mitochondria, endoplasmic reticulum NADPH oxidases, peroxisomes, and plasma membrane [3,4,12]. ROS are also produced in the nucleus, as transcription activation involves the demethylation of histones and 5-methyl cytosines in promoter CpG islands—two modifications that generate ROS as byproducts [12,13,14]. When nuclear ROS reach a certain level, they may damage DNA and impair cell function. Among the four bases, guanine, having the lowest redox potential, is oxidized to 8-oxoguanine (8OG) [15]. 8OG is considered the main product of oxidation, but it can be further oxidized to spiroiminodihydantoin (Sp) and guanidinohydantoin (Gh) [16]. As 8OG couples with cytosine or adenine, 8OG:C → T:A transversions occurring during DNA replication, increasing the risk of developing cancer. Oxidized guanine is recognized in the cells by DNA glycosylases, i.e., enzymes involved in the base excision repair pathway (BER). Two important glycosylases that have been extensively studied are 8-oxoguanine glycosylase (OGG1) and endonuclease VIII-like 1 (Neil1) [17,18]. 8OG is frequently present in clusters of guanines, including G-quadruplex (G4) motifs. One well studied G4-motif is located in the *Kirsten ras* (*KRAS*) promoter upstream the transcription start site. The *KRAS* gene is mutated in >90% pancreatic ductal adenocarcinoma (PDAC) and ≈50% colorectal adenocarcinomas [19,20]. Mutations in exon 1 at codons 12, 13, or 61 of the *KRAS* gene inhibit the GTPase activity of the RAS protein, which remains locked in the activated GTP-bound state, stimulating constitutively survival and proliferation pathways [21]. Recent research has shown that *KRAS* reprograms both glucose and glutamine metabolism to produce biomass for feeding a high rate of cell divisions [22,23]. Since ROS are enhanced in pancreatic cancer cells [6,7,8,24], they express a high level of Nrf2 to maintain the redox homeostasis [25]. Nevertheless, compared to non-cancer cells, they show a higher level of 8OG [26]. As 8OG is a mutagenic base lesion, it is repaired by the BER system of the cells [27,28,29,30], and glycosylase OGG1 is a primary enzyme for the excision of 8OG from duplex DNA. Sp and Gh lesions are instead efficiently excised by glycosylase Neil1 [31]. However, it has been found that Neil1 is able to remove 8OG in proximity to the 3′ end of a single-stranded break, where OGG1 shows reduced activity [32]. Moreover, Neil1 excises 8OG from DNA bubbles and DNA double strands, generating β, δ-elimination products [33,34]. As the expression of the *KRAS* gene is regulated by a G4 motif prone to oxidation, called 32R [35,36], in this study, we asked if OGG1 and Neil1 are able to excise 8OG from duplex and G4 DNA conformations. We provide evidence that OGG1 efficiently excises 8OG from duplex but not from G4 DNA, whereas Neil1 strongly recognizes G4 DNA and cleaves the structure with an efficiency depending on G4 topology. The results of our study may have implications for the mechanism of *Kirsten ras* (*KRAS*) transcription.

## 2. Results and Discussion

### 2.1. Structure of Wild-Type KRAS G4 and Oxidized G4 Analogues

**Figure 1:**
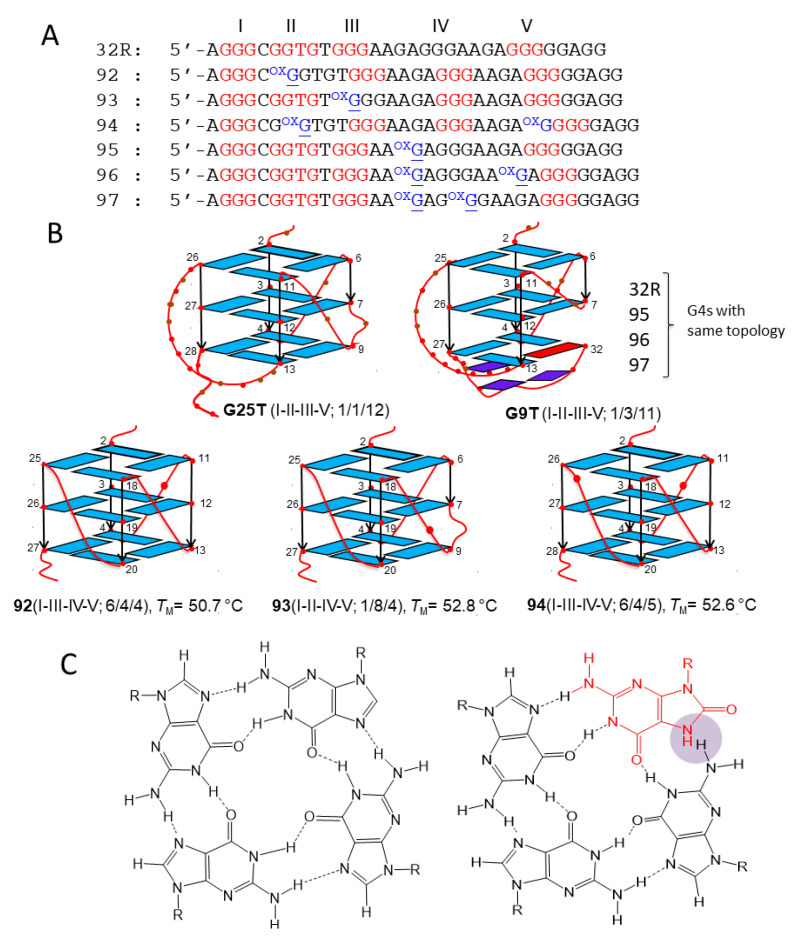
(**A**) Sequence of wild-type 32R and of its 8-oxoguanine (8OG)-containing analogues **92**–**97**. In sequences **92**–**94**, 8OG is located in a G-run, while in sequences **95**–**97**, 8OG is located in the major 11(12)-nucleotide (nt) loop. The bases in red form the three G-tetrads of the G-quadruplex (G4). (**B**) NMR structures of the 32R conformers G25T and G9T and DMS footprinting structures of the G4s formed by oxidized **92–94.** In G9T, the bases in dark blue (G28, A30, G31) form a triad at the 3′-end that contributes to stabilizing the G4; the base in red is a guanine in *syn* [38]. (**C**) Structure of normal and oxidized G-tetrads. 8OG destabilizes a G4 as its N7, becoming a donor of H-bond clashes with NH2 of a neighboring guanine.

32R is located in the *KRAS* promoter, upstream of the transcription start site (TSS). It is composed by 5 G-runs (I-V) folding into a stable G4 structure [35,37] (Figure 1A,B). Initial footprinting experiments showed unambiguously that the G-runs I-II-III-V form the scaffold of the four-stranded structure [35]. Subsequent NMR experiments revealed that 32R folds in two conformers with different structural features that are in slow equilibrium with each other: conformer G25T, with a T-bulge in one strand, all guanines in *anti*-conformation, and 1/1/12 loops; conformer G9T with a fold-back guanine in *syn*, a three-base triad at the 3′ end, and 1/3/11 loops [38] (Figure 1B).

Sequence 32R is recognized by several nuclear factors including MAZ, hnRNP A1, Ku70, and PARP1 [35]. Preliminary data have shown that both conformers are recognized by the nuclear factors, albeit to different extents (in preparation). Evidence that 32R behaves as a regulatory element for *KRAS* transcription has been provided [35,37,39,40]. The mechanism by which the gene is regulated is rather complex and recent data suggest that guanine oxidation to 8OG might play a role, in particular under-enhanced oxidative stress, which is typical of cancer cells [26,39]. This is based on the following experimental observations: (i) guanines in 32R tend to oxidize more than isolated guanines; the guanine at the 5′ end of a G-run shows the lowest ionization potential, i.e., the highest tendency for oxidation [41,42,43]; (ii) the *KRAS* gene is responsive to ROS, as it is upregulated when the cellular oxidative stress is increased by H_2_O_2_ or by photoactivated porphyrins [39,44]; (iii) pulldown and chromatin immunoprecipitation (ChIP) experiments show that 8OG and G4 co-localize in the same promoter region containing 32R [26]. Together these findings suggest that the 32R motif located in the *KRAS* promoter, upstream TSS, is a sensitive site for guanine oxidation. To examine the impact of 8OG on 32R, we designed oligonucleotides with 8OG lesions either in a G-run forming the G4 core (sequences **92–94**) or in the major loop (sequences **95–97**) (Figure 1A). It should be remembered that 8OG destabilizes a G-tetrad, as N7 of the purine ring becomes a donor of H-bond (Figure 1C). We have previously demonstrated by dimethyl sulfate (DMS) footprinting that sequences **92–94** fold differently from wild-type 32R, as the G-run bearing 8OG is replaced by the extra G-run present 32R. Thus, **92, 93,** and **94** assume a G4 with a 6/4/4, 1/8/4, and a 6/4/5 topology, respectively [26] (Figure 1). Their *T*_M_’s are roughly 10 °C lower than that of wild-type G4 (50.7, 52.8, 52.6, and 62.1 °C for **92**, **93**, **94**, and 32R, respectively). By contrast, sequences **95–97**, with 8OG lesions in the major loop, maintain the same 1/1/11(12) topology of wild-type 32R and are characterized by thermal stabilities similar to that of wild-type 32R (61.5, 63.7, and 62.1 °C for **95**, **96**, and **97**) [26]. 

### 2.2. The 32R KRAS Motif in Duplex Conformation Was Recognized by Glycosylases OGG1 and Neil1

Recombinant glycosylases OGG1 and Neil1 were obtained as previously described [26]. We started our study by comparing the capacity of the two glycosylases to excise 8OG from wild-type and oxidized double-stranded 32R. The designed oligonucleotides bearing 8OG (sequences **92**–**97**) and wild-type 32R (see Figure 1A) were labelled at the 5′ end with [γ-^32^P]ATP, mixed with the complementary strand and annealed in duplexes.

**Figure 2:**
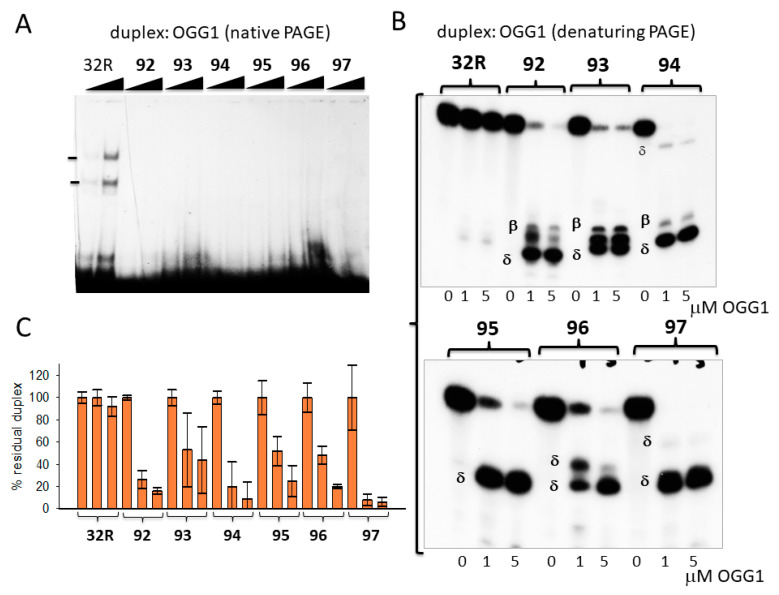
(**A**) Native 5% PAGE in Tris/borate/ethylenediaminetetraacetic acid (EDTA) (TBE) at 20 °C of mixtures containing 8 nM radiolabeled wild-type and oxidized duplexes and 8-oxoguanine glycosylase (OGG1) (1 and 5 μg) in buffer B (see Materials and Methods). (**B**) Denaturing 20% PAGE in TBE at 55 °C showing the integrity of wild-type and oxidized duplexes after 15 min incubation with 0, 1, and 5 μM OGG1 in buffer A. The expected β and δ elimination products are indicated. (**C**) Bar plots showing the percentage residual duplex after 15 min digestion of the designed duplexes with OGG1. Error bars were obtained from three experiments.

We incubated the duplexes (8 nM) with 1 and 5 μg OGG1 in binding buffer (buffer A, electrophoresis mobility shift assays (EMSA), Section 3.5) for 15 min and run the mixtures in a native 5 % PAGE (Figure 2A). Only the wild-type 32R duplex and not the oxidized analogues (**92**–**97**) showed two retarded bands, most likely due to the formation of 1:1 and 1:2 DNA/protein complexes. We rationalized this behavior, assuming that the cleavage by OGG1 of the oxidized duplexes destabilized the complexes. In fact, when the mixtures, after 15 min incubation in buffer B at 37 °C, were run in a denaturing 20 % PAGE, allowing us to detect the integrity of the duplexes after incubation with OGG1, we found that the sequences were indeed cleaved. It should be borne in mind that OGG1 and Neil1 are bifunctional DNA glycosylases—besides removing an oxidized base from duplex DNA, which creates an apurinic/apyrimidinic (AP) site, they also cleave the phosphodiester backbone and generate β, δ-elimination products (AP lyase ractivity) [45]. Figure 2B shows that OGG1 does not have effect on the wild-type duplex, as expected, while it efficiently excises 8OG and cleaves the backbone of the oxidized analogues. Duplexes **92**, **93**, and **94**, with 8OG in the 5′-half of the sequence, gave, respectively, the 6-, 11-, and 7-mer β-elimination fragments and 5-, 10-, and 6-mer δ-elimination fragments. Duplexes **95–97**, with 8OG in the 3′-half of the sequence, gave only δ products. Duplex **95** gave the expected 15-mer δ-fragment, while duplexes **96** and **97,** with two 8OGs, gave the expected 15-mer/18-mer and 15-mer/22-mer δ-fragments, respectively. We reported in a bar plot the percentage residual duplex after a treatment with 0, 1, and 5 μM OGG1 for 15 min (Figure 2C). Note that the wild-type duplex was not affected by the glycosylase, but the oxidized analogues were nearly completely cleaved (% residual duplex < 20%), thus confirming OGG1′s efficiency in excising 8OG from duplex 32R. 

**Figure 3:**
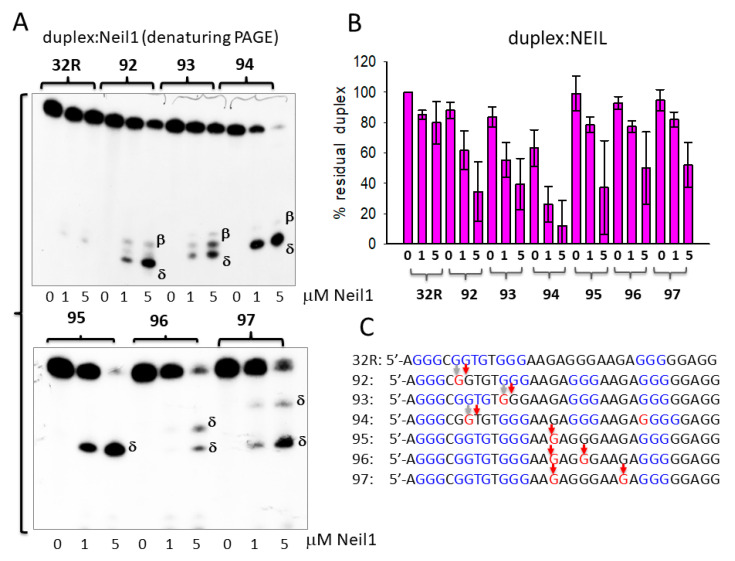
(**A**) Denaturing 20% PAGE in TBE showing the products obtained by incubating the designed wild-type and oxidized 32R duplexes with endonuclease VIII-like 1 (Neil1) (0, 1, 5 μM). Band of β- and δ-elimination products are indicated. (**B**) Bar plot showing the percentage residual duplex after 30 min incubation of the designed duplexes with 0, 1, and 5 μM Neil1. (**C**) Cleavage sites of Neil1 on the designed 8OG-containing duplexes.

In Figure 3A–C, we report the results obtained with glycosylase Neil1. Again, Neil1 did not have an effect on the wild-type duplex, whereas it cleaved the oxidized analogues, although to a lesser extent than OGG1. Indeed, while 1 μM OGG1 efficiently cleaved duplexes **92**, **93**, **94**, **95**, **96**, and **97** with percentage residual duplex values of ≈ 20, 50, 20, 50, 50, and 10 %, respectively**,** 1 μM Neil1 showed a lower catalytic activity, with percentage residual duplex of ≈ 60, 60, 30, 80, 80, and 90% for the same **92**–**97** duplexes, respectively. Neil1 cleaves the duplexes at the site of 8OG, producing β and δ elimination products, as observed with OGG1. The data suggest that the glycosylases cleaved the duplexes exactly in correspondence of the oxidized base (Figure 3C). Together, the results show that 8OG in the designed duplexes was excised and the DNA backbone was cleaved by Neil1, although less efficiently than OGG1.

### 2.3. Activity of Glycosylase Neil1 and OGG1 on the 32R KRAS Motif in G4 Conformation 

**Figure 4:**
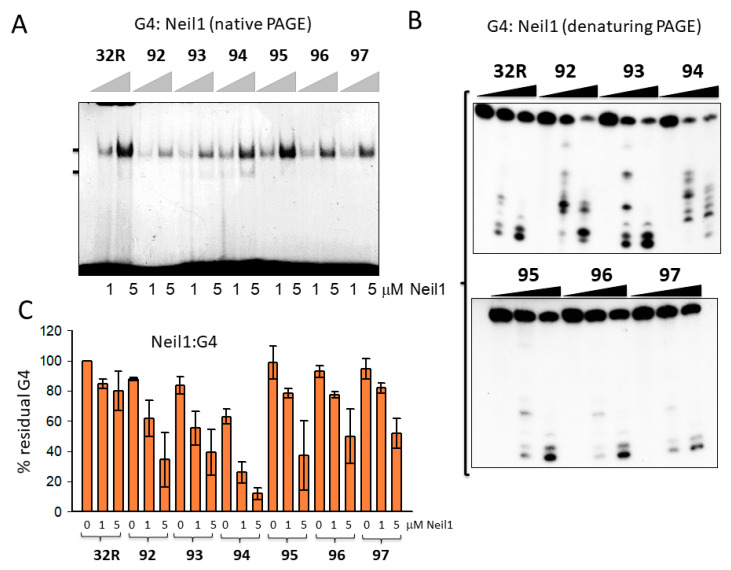
(**A**) Native 5% PAGE in TBE of mixtures containing 8 nM radiolabeled wild-type and oxidized 32R G4s and 1–5 μg Neil1 (15 min incubation in buffer B). (**B**) Denaturing 20% PAGE in TBE showing the cleavage activity of 0, 1, and 5 μM Neil1 on wild-type and oxidized 32R G4. (**C**) Bar plots showing the percentage residual G4 after 30 min digestion with Neil1. Error bars have been obtained from two experiments.

Next, we focused on 32R in the folded G4 conformation and asked if OGG1 and Neil1 are able to excise 8OG also from this non-Watson-Crick (WC) substrate. To address this issue, we let the radiolabeled oligonucleotides fold overnight in a buffer containing 50 mM Tris-HCl (pH 7.4) and 100 mM KCl, then we incubated them in buffer B for 15 min with 1 and 5 μg of recombinant OGG1 or Neil1 and run them in native 5% PAGE. Figure 4A shows that Neil1 bound to the designed G4 structures, forming a complex migrating in the gel with a retarded band of intensity depending on the topology of the G4 structure. Note that **92** and **93** show a retarded band weaker than that of wild-type 32R. This may point to a weaker binding or, alternatively, that Neil1, upon binding, cleaves the G4 and destabilizes the DNA–protein complex. We previously reported [26] that OGG1 binds to the oxidized **92**–**97** G4s too, but in this case the retarded bands of the complexes showed similar intensities. This suggests that OGG1 does not cleave the G4s, as demonstrated in a denaturing gel and in agreement with the results by Zhou et al. [31]. To further investigate the activity of Neil1 on the designed G4s, we incubated the protein with the G4s and analyzed the products in 20% denaturing PAGE, which allowed us to determine the integrity of the G4 sequences after exposure to Neil1 (Figure 4B).

**Figure 5:**
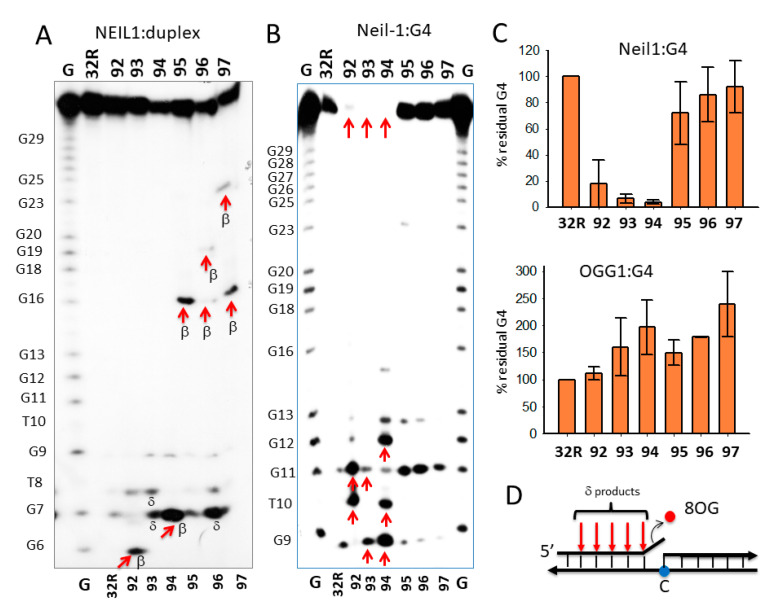

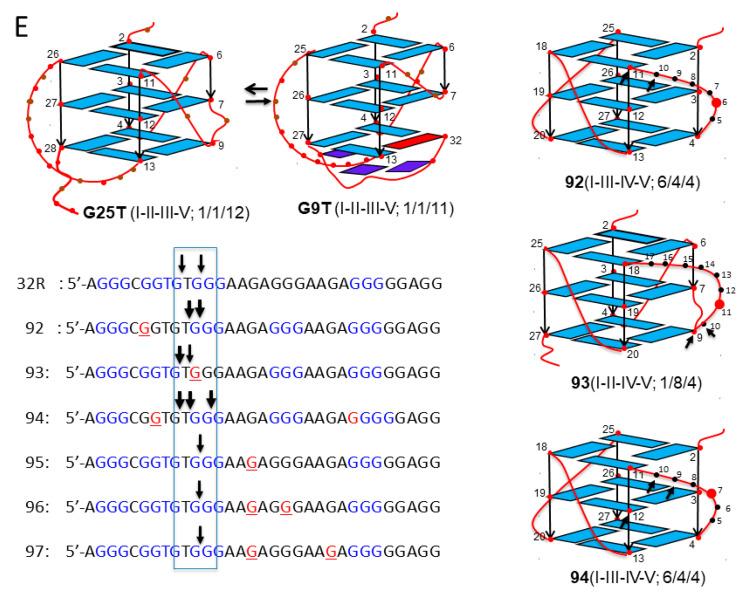
(**A**,**B**) Sequencing 20% PAGE showing the cleavage activity of Neil1 on wild-type and oxidized 32R in duplex (**A**) and G4 (**B**) conformations. Arrows and letters in (**A**) indicate the expected bands according to the position of 8OG within the duplex and the type of cleavage. In (**B**), we indicate with arrows the major bands obtained from the **92–94** G4s that were completely cleaved. The **95–97** G4s were cleaved at G11. (**C**) Bar plots reporting the percentage residual G4 after 15 min incubation with 5 μM Neil1 and OGG1 (not shown as OGG1 did not cleave any G4). Data are from three experiments. (**D**) Scheme showing δ-elimination products involving fragments with one or more nucleotides. (**E**) Structures of wild-type and oxidized G4**.** In G9T, the bases in dark blue (G28, A30, G31) are for a triad at the 3′-end that contributes to stabilizing the G4; the base in red is a guanine in *syn* [38]. The arrows show the position of Neil1 cleaving sites. The sequences of the oxidized G4s with arrows showing the Neil1 cleaving sites are also reported.

The results obtained demonstrated that wild-type 32R and oxidized **95–97** adopting the same 1/1/11(12) structures show a similar digestion pattern, independently from the presence or absence of 8OG in the G4. These G4s were slightly cleaved at G11 (as indicated by the sequencing gel of Figure 5B). This shows that Neil1 is characterized by a weak 8OG-independent cleavage activity against G4 DNA (but not against duplex 32R, see Figure 3A). By contrast, sequences **92–94**, forming G4s with a different topology, 6/4/4, 1/8/4, and 6/4/5, were strongly cleaved by Neil1. In Figure 4C we report the percentage residual G4 after incubation of the G4s with increasing amounts of Neil1. It can be seen that **92–94** were strongly cleaved after 15 min incubation with 5 μM Neil1, the percentage residual G4s were ≈ 30, 40, and 10%, respectively. 

To further investigate the cleavage activity of Neil1 on the designed oligonucleotides in duplex and G4 conformation, we analyzed the products of the digestion in a 20% polyacrylamide sequencing gel (Figure 5). To rationalize the results of the PAGE analysis, it should be considered that the activity of bifunctional Neil1 DNA glycosylase involves three steps: (i) excision of the oxidized base with the formation of an apurinic/apyrimidinic (AP) site; (ii) cleavage of the DNA backbone by β-elimination, yielding two fragments, one of which contains an unsaturated sugar moiety; and (iii) removal of the unsaturated sugar from one of the two fragments via δ-elimination. Figure 5A shows the digestion products of wild-type and oxidized 32R duplexes by Neil1. In lane 1, we report a G-reaction with DMS/piperidine of the G-rich 32R sequence. The bands marked with an arrow indicate the expected DNA fragment if Neil1 cleaves the duplexes exactly in correspondence of 8OG. It is worth noting that duplexes **95–97**, bearing 8OGs in the middle and 3′-half of the sequence, gave the expected bands from a β-elimination activity (i.e., the cleavage of the backbone at the 3′ side of 8OG): a 16-mer fragment from **95**, 16- and 19-mer fragments from **96**, and 16- and 23-mer fragments from duplex **97**. Duplex **96** with two 8OG lesions close one another also gave a shorter 7-mer fragment that could result from a δ-elimination of a fragment containing the unsaturated sugar and some nucleotides [45] (Figure 5D). Duplexes **92** and **94** gave the expected 6-mer and 7-mer fragments from β-elimination, respectively, while duplex **93** yielded fragments lower than the expected 11-mer, which may have been due to δ-eliminations involving more than one nucleotide. 

The analysis in a polyacrylamide sequencing gel of the digestion products was extended to the wild-type and oxidized 32R G4 structures (Figure 5B). It showed that the activity of Neil1 depended on the G4 topology—while **92–94** G4s were completely cleaved by the glycosylase (percentage residual G4 ≈ 0%), the G4 variants **95–97** were only slightly affected by Neil1. This result shows that the activity of Neil1 strongly depends on the topology of the G4 structures. In Figure 5E, we summarize the G4 structures adopted by the oxidized sequence on the basis of DMS footprinting [26]. The sequencing gel shows that (i) **92**, forming a 6/4/4 G4, was cleaved in the 6-nt loop containing 8OG at T10 and G11 (marked with arrows); (ii) **93**, forming a 1/8/4 G4, was instead cleaved at G9 and in the loop at G11; (iii) **94**, adopting a 6/4/5 G4, was cleaved in the 6-nt loop containing 8OG at G9/T10 and at G12/G13. Figure 5C shows the percentage residual G4 after treatment with Neil1 and OGG1. 

While OGG1 did not have any effect on the G4s (not shown), Neil1 efficiently cleaved the G4s with a topology different from 1/1/11(12). Previous studies have supported the notion that Neil1 is more specific for guanine oxidized to Gh and Sp, and for G lesions present in G4 DNA [31]. Here, we report that Neil1 is able to efficiently excise 8OG from *KRAS* G4. However, this activity strongly depends on the topology of the G4 structure. Surprisingly, we found that the wild-type 32R sequence folding in a G4 with a large loop of 1/1/12-nt is a weak substrate for Neil1. By contrast, the G4s with shorter loops, **92–94**, were efficiently cleaved by Neil1. It is noteworthy that while the duplexes showed a backbone cleavage at 8OG sites, the same sequences in G4 were cleaved by Neil1 at different sites in the neighborhood of 8OG. As reported in Figure 5E, the *KRAS* G4s were cleaved by Neil1 between G9 and G13. This is the first piece of evidence that Neil1 cleaves G4 DNA in a way that depends on the G4 topology. The fact that more than one band was obtained may have been due to the presence in solution of multiple G4 conformers formed by a given sequence. Indeed, this was not observed with duplex 32R, as it assumed a unique WC conformation. 

### 2.4. Possible Role of Glycosylase Neil1 in the KRAS Promoter

**Figure 6:**
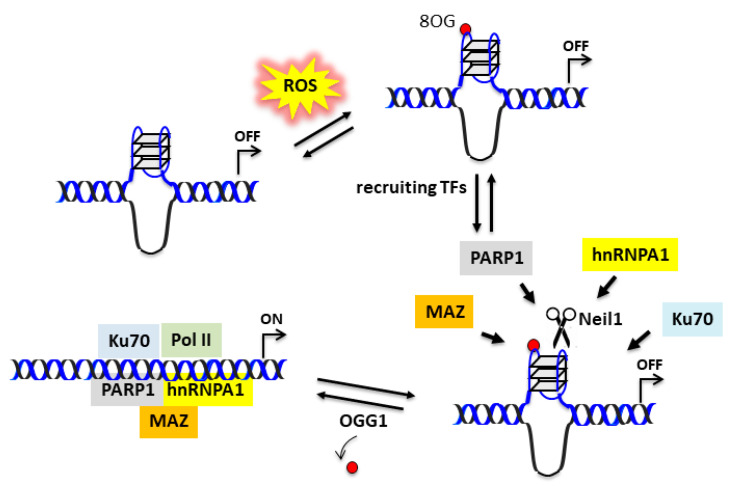
Possible function of Neil1 in upregulating the *KRAS* gene in the presence of enhanced oxidative stress. 8OG in 32R G4 can be regarded as an epigenetic mark stimulating the recruitment of transcription factors. Neil1 may cleave the G4 structure, thus favoring the unfolding initiated by MAZ or hnRNP A1. When 32R assumes the canonical duplex conformation, OGG1/Neil1 removes 8OG and the transcription pre-initiation complex is formed.

Experimental data suggest that the 32R motif located in the *KRAS* promoter is folded into a G4 under physiological conditions. The folding should be favored by DNA supercoiling, which provides the energy to locally unwind the promoter [46]. It is likely that the complementary C-rich strand assumes an *i*-motif [47,48,49]. As PDAC cells have a higher level of oxidative stress than normal cells [44], the G-runs of 32R are prone to oxidation because of the guanine’s low redox potential [42]. Indeed, a ChIP qPCR analysis showed that 8OG is more abundant in the promoter region containing 32R than in other G-rich genomic sequences, which are unable to fold into a G4 [26]. We found that oxidized 32R stimulated the recruitment to the promoter of nuclear factors involved in *KRAS* transcription: PARP1, MAZ, Ku70, and hnRNP A1 [35,39]. Besides being components of the transcription initiation complex, MAZ and hnRNP A1 have the capacity to unfold the G4 [50,51]. In the reconstituted duplex, 8OG is efficiently removed by OGG1 via the BER pathway [26]. The extension of 32R from G4 to duplex in the presence of negative supercoiling is not an easy process. However, the supercoiling energy can be released by introducing a nick in one strand of DNA. As depicted in Figure 6, glycosylase Neil1 by cleaving the G4 backbone could favor the transformation of 32R from the folded to the duplex state. 

## 3. Materials and Methods

### 3.1. Oligonucleotides 

Unmodified oligonucleotides were purchased from Microsynth (CH), while 8OG-substituted oligonucleotides were synthesized in a 1-μmol scale on solid support following a standard procedure and using 8-oxo-dG CEP from Berry and Associates. The deprotection step was carried out as described by Bodepudi et al. [52], i.e., using concentrated ammonia in the presence of 2-mercaptoethanol (0.25 M). The oligonucleotides were purified by reverse-phase high pressure (or high performance) liquid chromatography on a Water system 600, equipped with a C18 column (XBridge OST C18, 19 × 1000 mm, 5 μm). The oligonucleotide composition was confirmed by matrix-assisted laser desorption ionization time-of-flight (MALDI-TOF). 

### 3.2. Cell Cultures

Human pancreatic cancer cells (Panc-1) were maintained in exponential growth in Dulbecco’s modified Eagle’s medium (DMEM) supplemented with 100 U/mL penicillin, 100 mg/mL streptomycin, 20 mM L-glutamine, and 10% fetal bovine serum (Euroclone, I). The cell line genotype, performed by Microsynth (CH), confirmed their identity.

### 3.3. Recombinant OGG1 and Neil1 Proteins

The recombinant OGG1 and Neil1 proteins with His-tag at the *N*-terminus were expressed in *Escherichia coli* (Novagen’s Rosetta host strain) transformed with plasmids pET20 hOGG1 or pET30-Neil1. The competent bacteria were grown for 2 h at 37 °C up to an absorbance of 0.8–1 units at 600 nm, prior to induction with IPTG (isopropyl 1-thio-β-d-galactopyranoside) (0.4 mM final concentration for OGG1, 1 mM for Neil1). The cells were allowed to grow overnight at 20 °C (Neil1) or 25 °C (OGG1) before harvesting. Then, the cells were centrifuged at 4 °C (5000 rpm), the supernatant was removed, and the pellet was resuspended in Lysis buffer (50 mM NaH_2_PO_4_, 300 mM NaCl and 10 mM imidazole (OGG1), 5 mM imidazole (Neil1)) added with 0.2 mM PMSF (phenylmethylsulphonyl fluoride). The bacteria were lysed by sonication (3 cycles of 30 s sonication/1 min off) and treated with 0.05% Tween 20 (Sigma-Aldrich, MO, USA). The lysate was centrifuged for 10 min at 4 °C, 10,000 rpm. The His-tagged proteins were purified by using Ni-nitrilotriacetic acid (NTA) resin (Qiagen, D), which was added to the supernatant, and the mixture was shaken for 1 h at 4 °C. After centrifugation for 5 min at 1700 rpm, the resulting pellet was washed 2 times with Wash Buffer (50 mM NaH_2_PO_4_, 300 mM NaCl and 20 mM imidazole for OGG1 or 5 mM imidazole for Neil1). The OGG1 or Neil1 protein bound to the resin were eluted with 50 mM NaH_2_PO_4_, 300 mM NaCl, and 600 mM imidazole. Protein concentration was determined by a Bradford assay, while the purity was confirmed by SDS-PAGE. Finally, the proteins were concentrated, desalted using a Ultracel YM-3 Microcon Centrifugal Filter Device (Millipore, MA, USA).

### 3.4. Analyses of DNA–Protein Mixtures in Sequencing Polyacrylamide Gels 

The unmodified and 8OG-modified oligonucleotides were first purified by denaturing PAGE, then end-labeled with [γ-^32^P] ATP and T4 polynucleotide kinase (30 pmol) (Thermo Fisher Scientific, Walthman, MA USA). The radiolabeled oligonucleotides were annealed in duplex as follows. The oligonucleotides were heated for 5 min at 95 °C and let to anneal overnight at room temperature in 50 mM Tris-HCl (pH 7.4) and 100 mM NaCl buffer containing the complementary strand. The annealing in quadruplex was carried out in 50 mM Tris-HCl (pH 7.4) and 100 mM KCl. Radiolabeled duplex or quadruplex (8 nM) were incubated at 37 °C with increasing amounts of OGG1 or Neil1 (0, 1, and 5 μM), in 20 mM Tris-HCl (pH 8), 50 mM NaCl (or 50 mM KCl for the quadruplex), 1 mM ethylenediaminetetraaceticacid (EDTA), 0.1 mg/mL bovine serum albumin, 1 mM Na_3_VO_4_, 5 mM NaF, and 0.01% phosphatase inhibitor cocktail (buffer B). The reactions were stopped after 15 min by adding to the mixtures 8 μL stop solution (90% formamide, 50 mM EDTA). The mixtures were then heated at 95 °C for 5 min and run for 1 h on a denaturing 20% PAGE prepared in Tris/borate/EDTA (TBE) and 7 M urea, which was pre-equilibrated at 55 °C in an electrophoretic apparatus (C.B.S Scientific Company, Del Mar, CA USA). After running, the gel was fixed in a solution containing 10% acetic acid and 10% methanol, dried, and exposed to auto-radiography (Aurogene, I).

### 3.5. Electrophoresis Mobility Shift Assays

Protein–DNA interactions were analyzed by native electrophoresis mobility shift assays (EMSA). Radiolabeled duplexes or quadruplexes (obtained as described above) were incubated for 15 min in 20 μL solutions containing 50 mM Tris-HCl (pH 7.4), 100 mM NaCl (for duplexes), or 100 mM KCl (for G-quadruplexes), 1 mM EDTA, 0.01% phosphatase inhibitor cocktail I (Sigma-Aldrich, MO, USA), 5 mM NaF, 1 mM Na_3_VO_4_, 2.5 ng/μL poly [dI−dC], 1 mM dithiothreitol (DTT) (buffer A), and 8% glycerol with increasing amounts of recombinant OGG1 or Neil1 (0, 1, and 5 μM). The DNA–protein mixtures were analyzed in a native 5% PAGE, as well as in 20% denaturing PAGE, in TBE at 20 °C. After running, the gels were dried and exposed overnight to auto-radiography (Aurogene, I) at −80°C.

## 4. Conclusions

Cancer cells with a high metabolic rate produce more ROS than normal cells [6]. Under oxidative stress, the guanines in G4 motifs are inclined to oxidize to 8OG due to their low redox potential [13]. Previous studies have shown that 8OG in 32R, the G4 motif in *KRAS* promoter critical for transcription, behaves as an epigenetic mark for the recruitment of transcription factors [26,39]. However, 8OG is mutagenic and is repaired by the cell. Here, we investigated the capacity of glycosylases OGG1 and Neil1 to excise 8OG from 32R, either in duplex or G4 conformation. We found that OGG1 efficiently excised 8OG from 32R in duplex but not in G4 conformation. In contrast, glycosylase Neil1 showed more activity against the G4 than the duplex conformation. By using a high-resolution polyacrylamide gel, our data showed that 8OG in 32R duplex was excised, although to a different extent, by both OGG1 and Neil1, and the phosphodiester backbone was cleaved with the production of the expected β and δ-elimination fragments. Interestingly, our data showed that the activity of Neil1 on folded 32R strongly depended on the topology of the G4 structures. This was in agreement with previous findings showing that Neil1 efficiently repaired hydantoin lesions from the parallel telomeric G4s, but not from the parallel G4 formed by the *VEGF* or *CMYC* motifs [53]. However, when the fifth G-run of the VEGF motif was incorporated in the sequence, Neil1 efficiently removed the lesion because the G4 changed the topology, thus becoming a substrate for Neil1 [31,54,55]. These experiments indicated that the activity of Neil1 on G4 DNA strongly depends on G4 topology. The data of this study may have implications for the mechanism activating transcription.

## Data Availability

The data presented in this study are available on request from the corresponding author.

## Abbreviations

G4	G-quadruplex
8OG	7,8-dihydro-8-oxoguanine
KRAS	Kirsten ras
OGG1	8-Oxoguanine glycosylase
Neil1	Endonuclease VIII-like 1
